# Total injectable anesthesia of dogs and cats for remote location veterinary sterilization clinic

**DOI:** 10.1186/s12917-020-02525-x

**Published:** 2020-08-24

**Authors:** Lysa Pam Posner, Jeffrey Applegate, Allen Cannedy, Diane Deresienski, Kristie Mozzachio, Maria Serrano, Gregory Lewbart

**Affiliations:** North Carolina State University, College of Veterinary Medicine, 1060 William Moore Drive, Raleigh, NC 27606 USA

**Keywords:** sterilization, dog, cat, anesthesia, total injectable anesthesia

## Abstract

**Background:**

Sterilization clinics often occur in remote places where anesthesia machines and compressed oxygen are unavailable. This study describes the use of total injectable anesthesia in dogs and cats presented for sterilization in a remote location.

**Results:**

A total of 100 animals were sterilized; 26 female cats (CF), 22 male cats (CM), 28 female dogs (DF), and 24 male dogs (DM). CF were anesthetized with dexmedetomidine (20 mcg/kg), ketamine (8 mg/kg) and hydromorphone (0.1 mg/kg) IM. CM were anesthetized with dexmedetomidine (15 mcg/kg), ketamine (5 mg/kg) and hydromorphone (0.1 mg/kg) IM. Insufficient anesthesia in cats was treated with alfaxalone (1 mg/kg) IM. All cats were administered meloxicam at 0.3 mg/kg SQ. DF were anesthetized with dexmedetomidine (15 mcg/kg), ketamine (7–10 mg/kg) and hydromorphone (0.1 mg/kg) IM. DM were anesthetized with dexmedetomidine (15 mcg/kg), ketamine (5 mg/kg) and hydromorphone (0.1 mg/kg) IM. All dogs had IV catheter and endotracheal tube placed. If SpO_2_ < 91%, ventilation was assisted with an Ambu bag. Insufficient anesthesia in dogs was treated with alfaxalone (1 mg/kg) IV. All dogs were administered meloxicam at 0.2 mg/kg SQ. Following surgery, atipamezole (0.05–0.1 mg/kg) IM was administered to any patient that did not have voluntary movement. All patients survived and were discharged. Less than 25% of cats and male dogs required supplemental anesthesia. Fifty seven percent of female dogs required supplemental anesthesia. More than 89% of patients (in any group) required atipamezole administration. One cat recovered with agitation and hyperthermia (41.1C/ 106F). Some dogs required ventilatory assistance to remain normoxemic while anesthetized.

**Conclusion:**

Total injectable anesthesia can be accomplished for remote location sterilization clinics with minimal morbidity.

## Background

Unwanted reproduction in dogs and cats negatively affects communities by contributing to: the spread of disease, aggression to humans, nuisance behavior (e.g. getting into garbage) and predation of other species (e.g. wild birds). Sterilization of both pet and feral dogs and cats is often needed in remote or underdeveloped areas, where access to veterinary medicine is limited or absent. Spay-neuter programs are offered in a variety of styles in these locations via mobile clinics, but access to facilities and equipment is variable. The Association of Shelter Veterinarian has offered guidelines to assure consistent care to dogs and cats presented to these clinics [1, 2]. However, these guidelines assume access to equipment that might not be available in some remote locations, such as compressed gasses (e.g. oxygen) or anesthesia machines [1, 2].

The Galapagos Islands are an archipelago off the coast of Ecuador. While renowned for their interesting and diverse wildlife, many of the islands have large populations of intact dogs and cats that predate indigenous and endangered wildlife species (e.g. birds, turtles, marine iguanas) [3–6]. Isabela Island is the largest of the Galapagos Islands, but has a population of only about 1800 people [7], and is accessible primarily by boat. Therefore, on islands such as this, getting all the equipment necessary for a sterilization clinic presents a significant logistical issue. From the planning stages, it was considered impractical to transport an anesthesia machine and/or oxygen tanks (without the ability to refill them) to Isabela Island. Therefore, by necessity, the team was required to plan the clinic with use of injectable anesthetics only. While anesthesia for high volume, low cost spay/neuter clinics has been studied [8, 9], the majority of those studies were done in locations where resources are available if needed, even though not routinely used (e.g. anesthetic machines/oxygen). Conversely, there is a paucity of information regarding anesthesia management and complications in remote location spay/neuter clinics where rescue use of oxygen and/ or inhalant anesthetics are not available. The aim was to describe the use of total injectable anesthesia in dogs and cats presented for surgical sterilization in a remote location.

## Results

A total of 100 patients were sterilized. Temperature in the surgery and recovery rooms ranged from 79.2–83.4 F (26.2–28.8 C). Signalment and presence of pregnancy are presented in Table 1.

**Table 1:** Signalment and pregnancy status of all patients

Group	n	Reported Age (yr)	Pediatric (< 16 weeks)	Weight (kg)	Pregnant
Feline OVH	26	0.5 (.17–3)	10 (38%)	2.2 (1.1–3.5)	3
Feline Castration	22	1 (0.25–4.5)	6 (27%)	2.5 (1.2–4.5)	n/a
Canine OVH	28	1.3 (.25–7)	1 (4%)	12.5 (3–25)	2
Canine Castration	24	1.4 (.17–3)	5 (21%)	14.7 (2.3–25)	n/a

Age and weight are presented as median and range

All patients survived anesthesia and surgery and were discharged. None of the cats required ventilatory support via Ambu bag and endotracheal tube placement based on RR and Sp0_2_%. With assisted ventilation, all dogs remained normoxemic (SpO_2_ > 91%) during anesthesia. Time from anesthetic injection to start of surgery, surgery time, total patient time, and rescue drugs required are presented in Table 2.

**Table 2:** Duration of anesthesia, surgery, total clinic time, and rescue drugs

Group	n	Time from drug injection to start of surgery (min)	Surgery time (min)	Number of times additional drugs needed^a^	Total time in clinic (min)
Feline OVH	26	15 (7–35)	26 (12–46)	7/26 = 27% Alfaxalone IM *n* = 6 (once) Alfaxalone IM *n* = 3 (twice) Dexmedetomidine + ketamine IM n = 1 (once)	55 (35–120)
Feline Castration	22	11 (3–22)	2 (1–6)	1/22 (1.2%) Dexmedetomidine + ketamine IM n = 1 (once)	35 (14–69)
Canine OVH	28	24 (13–37)	34.5 (21–60)	16/28 (57%) Alfaxalone IV *n* = 12 (once) Alfaxalone IV n = 2 (twice) Alfaxalone IV *n* = 1 (thrice)	72 (56–107)
Canine Castration	24	25 (17–31)	18 (10–40)	2/24 (8%) Alfaxalone IV n = 2 (once)	58 (38–120)

Times are presented as median and range

^a^Rescue drugs were given when initial dosing was insufficient to maintain anesthesia

The number of patients administered atipamezole, the total amounts of atipamezole administered, time from atipamezole to recovery, time to recovery following atipamezole administration, and temperature at recovery are presented in Table 3.

**Table 3:** Reversal drug requirements, duration of recovery from reversal and recovery temperature

Group	n	Time from reversal to recovery (min)	Patients requiring reversal	Temperature at recovery (F/C)
Feline OVH	26	12 (3–60)	24/26 (92%) Atipamezole IM n = 24 (once)	99.1 (95.7–106.0) F 37.3 (35.4–41.1) C
Feline Castration	22	20 (4–50)	22/22 (100%) Atipamezole IM *n* = 21 (once) Atipamezole IM n = 1 (twice)	101.9 (99–102.7) F 38.8(37.2–39.3) C
Canine OVH	28	12 (4–30)	25/28 (89%) Atipamezole IM *n* = 24 (once) Atipamezole IM n = 1 (twice)	102.6 (97.3–104.1) F 39.2 (36.3–40.1) C
Canine Castration	24	14 (8–83)	22/24 (92%) Atipamezole IM *n* = 20 (once) Atipamezole IM n = 2 (twice)	102.3 (101.2–104) F 39.1 (38.4–40.0) C

Time and temperature are presented as median and range

Following OVH, one of the cats had an agitated recovery and was hyperthermic (106F/41.1C). The cat was monitored during recovery and both the hyperthermia and agitation resolved without treatment. The first three dogs castrated were administered ketamine at 7 mg/kg and demonstrated dysphoria following recovery from anesthesia. At the direction of the anesthesiologist, subsequent dogs had ketamine dosage reduced to 5 mg/kg and no other dogs demonstrated dysphoric recoveries. Three dogs having an OVH had dysphoric recoveries and were treated with 0.01 mg/kg acepromazine IV (Acepromazine, Vedco Inc., Saint Joseph Missouri) which resolved the dysphoria. Two dogs presented for castration demonstrated seizure-like activity during induction; muscle rigidity and muscle spasm. Seizure-like activity was treated in both dogs with alfaxalone at 1–2 mg/kg IV; which stopped the muscle fasciculations, and muscle relaxation occurred. Both dogs were castrated and recovered uneventfully. The remaining dogs and cats recovered well, with no overt signs of pain/discomfort. Dogs that underwent OVH were discharged with carprofen at approximately 4 mg/kg PO SID for 3 days.

Antibiotics and anthelminthics were provided for many patients at discharge, but that data is outside the scope of this paper.

## Statistics

Descriptive statistics are presented a median and ranges (min-max).

## Discussion

The data presented supports the hypothesis that anesthesia for spay/castration clinics can be accomplished in remote locations where anesthesia machines and/or oxygen is not available. Furthermore, this study has evaluated anesthetic protocols that produced rapid anesthesia, but allowed for reversal and rapid recovery of patients. This is important in many remote locations, as there are often few if any recovery cages/ holding areas, some of the patients are feral, and the longer the patient remain in the clinic’s care, the less patients that can be seen per day. Total patient time in the clinic was approximately 1 h, with cat castrations requiring the shortest stay and dog OVH requiring the longest.

Surgery time ranged from 1 min to 1 h. This made estimation of IM drug dosages needed difficult. Not surprisingly, 1% of cats being castrated needed rescue anesthetics while 57% of dogs undergoing an OVH required additional anesthetics. In cats that were insufficiently anesthetized during surgery, alfaxalone IM provided a rapid deepening of anesthesia. Intramuscular alfaxalone has been evaluated in cats for physiologic stability and PK/PD profiles [10, 11]. When administered to cats at 5 mg/kg IM, cats showed good physiologic stability, but recovery quality was considered poor [10]. In a different study, intramuscular alfaxalone at the same dosage was shown to reach peak concentration (Tmax) in ~ 22 min [11]. The cats in this study did not demonstrate any unpleasant recovery characteristics, likely due to the smaller dosage used in this study (1.0 mg/kg IM). Additionally, intramuscular alfaxalone worked rapidly enough to be considered a good choice for rescue anesthesia during a surgical procedure in a cat. Based on the Tmax of ~ 22 min, it was unexpected, but repeatable that the dosage and route was sufficient. The success of the dosage and route of alfaxalone in cats was likely due to the robust dosages of the induction agents used. For dogs that were inadequately anesthetized for surgery, alfaxalone IV provided suitable conditions for canine OVH and castration maintenance of anesthesia. This agrees with data showing that following premedications, a constant rate infusion of alfaxalone produced suitable anesthesia conditions for dogs undergoing OVH [12]. However, in both the Suarez study as in this study, many of the dogs required assisted ventilation to remain normoxemic [12].

Due to the lack of boarding space, and the potential for patients to be unsupervised outside, any animal that was not able to walk was administered atipamezole. While the loss of analgesia was considered, there is evidence in cats that administration of atipamezole did not negatively affect post-operative analgesia in cats that also received an opioid and ketamine as was used in this study [13]. Additionally, all the animals received an NSAID to supplement analgesia. The vast majority of patients required the antagonism of dexmedetomidine with atipamezole (89–100% of patients). In the first three dogs that were castrated, the dogs became agitated/dysphoric after atipamezole administration. Ketamine at anesthetic dosages are associated with a high incidence of agitation in the recovery period in humans and veterinary patients [14, 15]. Anesthesiologists often combine administration of ketamine with other CNS depressants such as benzodiazepines and alpha-2 adrenergic agonists to balance the risk of an agitated recovery from anesthesia [15]. Thus, the most likely reason for the agitation following administration of atipamezole in those dogs was loss of CNS depression from dexmedetomidine, which was balancing the behavioral effects of ketamine in the relatively short castration surgery. Immediately following the three dysphoric dog neuter recoveries, the anesthesiologist decreased the dosage of ketamine from 7 mg/kg to 5 mg/kg and none of the subsequent neuters had dysphoric recoveries. The majority of patients only required one dose of atipamezole with four patients requiring second doses (feline neuter (*n* = 1), canine OVH (n = 1), canine neuter (*n* = 2). Following OVH, three dogs demonstrated dysphoric recoveries. All three dogs were treated with acepromazine which successfully calmed them. In retrospect, it might have been possible to decrease the ketamine dose in OVH dogs and increase the dosage of alfaxalone for intraoperative maintenance. However, of the 28 canine OVH performed, only three had dysphoric recoveries (11%). None of the dogs or cats required additional analgesia before discharge. As expected, the combination of an alpha-2 agonist, an opioid, and a NSAID provided appropriate analgesia.

Body temperature during recovery ranged from 95.7–106 F (35.4–41.1 C). Inspection of Table 3 showes that the majority of patients remained normothermic. This was likely due to the combination of a warm surgery /recovery environment, the lack of cold, dry, anesthetic gases, and the rapid time of surgery/ anesthesia. One cat did become significantly hyperthermic (106F, 41.1C). Hydromorphone has been implicated in post anesthesia hyperthermia in cats [16], but it is unclear in this cat if the agitation was the cause or the result of the hyperthermia.

Two dogs being castrated had seizure-like activity during IM induction of anesthesia. Both dogs showed convulsive type behavior with loss of responsiveness, but neither dog became incontinent during the episode. Ketamine administration does enhance seizure like electroencephalogram waveforms [17], and it is has been implicated in causing seizures in a variety of veterinary species [15]. Therefore it is possible that both dogs did have seizures following high dose ketamine administration. However, it is also possible that unbalanced absorption of ketamine and dexmedetomidine following IM administration might have resulted in an exaggerated Stage 2 plane of anesthesia (involuntary excitement) which appeared seizure-like and the unresponsiveness was due to anesthesia induction. Both of those dogs muscle fasciculation/ contraction stopped after IV alfaxalone administration and both dogs recovered uneventfully.

Although supplemental oxygen was not available for the patients in this study, many routine procedures requiring anesthesia are performed outside of a veterinary hospital without incident (e.g. horse castrations, wildlife immobilization, spay/neuter clinics). Similarly, while endotracheal intubation of anesthetized cats is routinely performed, The Association of Shelter Veterinarians’ 2016 Veterinary Medical Care Guidelines for Spay-Neuter Programs indicated it is acceptable not to intubate cats for short duration surgeries (e.g OVH and castrations) as long as equipment for intubation is available in emergency situations [1]. Based on pulse oximetry, cats remained normoxemic thus none of the cats required intubation during anesthesia. Conversely, many dogs required assisted ventilation via an Ambu bag and endotracheal tube to maintain normoxemia. Placing and securing an endotracheal tube did not appreciably increase total procedure time and proved important in patients that required ventilatory support, particularly when supplemental oxygen was not available. While the extra safety of have supplemental oxygen is always preferable, this study demonstrated that the majority, if not all healthy dogs and cats can be appropriately anesthetized for the castration and OVH without supplemental oxygen, albeit they may require intubation and assisted ventilation.

A major limitation of this study is that it was not prospectively designed as a research project and thus some pieces of data were not consistently collected. However, it does provide valuable information regarding the use of total injectable anesthesia in dogs and cats, without rescue inhalants or oxygen. Physiologic assessment of dogs and cats anesthetized and sterilized with these protocols needs to be done. Additional limitations include: variability within each group in patient age and size, variability with four different surgeons, and varying health of patients. However, since this type of variability is expected in a remote-clinic setting, the overall success of the anesthetic plans is promising.

## Conclusion

Total injectable anesthesia can be accomplished for remote location sterilization clinics with minimal morbidity.

## Methods

In June of 2016 a team of 14 volunteers from the USA who all work within the veterinary community traveled to Isabela Island, Galapagos, to provide sterilization for any dog or cat (pet or feral). The team consisted of nine veterinarians, one licensed veterinary technician, two veterinary assistants, and two general assistants.

A local organization, the Intercultural Outreach Initiative, provided advertisement for the clinic as well as a building equipped with electricity and cold water. All equipment and supplies were transported by the volunteers via boat to the island.

Informed consent in writing was provided by the owners or guardians for all animals that were sterilized. At least one translator was present with each owner/guardian during the consent process. Following admission, each patient had a physical examination by an attending veterinarian, who decided if the patient was an appropriate candidate for anesthesia and sterilization. Each animal was weighed with a hanging luggage scale. Exclusion criteria included: presence of a fever, vomiting/diarrhea, emaciation, or severe cardiovascular disease. Anesthetic protocols were developed by a veterinary anesthesiologist (LPP) for each species and sex (Table 4). Dogs and cats were sterilized in order of arrival. Additional considerations for the protocols included behavior of feral patients, potential parasite burdens/occult disease, and the lack of anesthesia machines or medical oxygen.

**Table 4:** Anesthetic drug dosages

Procedure	Initial Anesthetic Drugs	Rescue Anesthetic Drugs	Adjunct Analgesics	Reversal
Cat OVH	Dexmedetomidine: 20 mcg/kg IM Ketamine: 8 mg/kg IM Hydromorphone: 0.1 mg/kg IM	Alfaxalone 1 mg/kg IM or Dexmedetomidine 5mcg/kg + ketamine 4 mg/kg IM	Meloxicam: 0.3 mg/kg SQ	Atipamezole 0.05 mg/kg IM
Cat Castration	Dexmedetomidine: 15 mcg/kg Ketamine: 5 mg/kg Hydromorphone: 0.05 mg/kg	Alfaxalone 1 mg/kg IM or Dexmedetomidine 5mcg/kg + ketamine 4 mg/kg IM	Meloxicam: 0.3 mg/kg SQ	Atipamezole 0.05 mg/kg IM
Dog OVH	Dexmedetomidine: 15 mcg/kg IM Ketamine: 7–10 mg/kg IM Hydromorphone: 0.1 mg/kg IM	Alfaxalone 1 mg/kg IV	Meloxicam: 0.2 mg/kg SQ	Atipamezole 0.1 mg/kg IM
Dog Castration	Dexmedetomidine: 15 mcg/kg IM Ketamine: 5–7 mg/kg IM Hydromorphone: 0.1 mg/kg IM	Alfaxalone 1 mg/kg IV	Meloxicam: 0.2 mg/kg SQ	Atipamezole 0.1 mg/kg IM

Alfaxalone, Alfaxan, 10 mg/ml, Jurox, Rutherford, NSW, Australia

Atipamezole; 5 mg/ml, Pfizer Animal Health, NY, NY, 10017

Dexmedetomidine hydrochloride, 500 mcg/ml; Pfizer Animal Health, NY, NY, 10017

Hydromorphone HCL, 1 mg/ml, Baxter Healthcare Corporation, Deerfield IL

Ketamine; 100 mg/ml, Fort Dodge Animal Health, Fort Dodge, Iowa, 50,501

Meloxicam 5 mg/ml, (Boehringer Ingelheim Vetmedica, St Joseph MO 64506

Following admission, each dog and cat were assigned a dedicated anesthetist from the time of premedication until recovery. Each animal was weighed with a hanging luggage scale and anesthetic drugs were calculated and dispensed by the anesthesiologist. Following IM drug administration each patient was assessed every 5 min for physiologic stability by monitoring HR, RR, mucus membrane color. Pulse oximetry (Sp0_2_) (Nonin 8500, Nonin Medical, Inc. Plymouth, Minnesota) was evaluated for each patient, but not consistently recorded. One oscillometric blood pressure (BP) monitor (Cardell 9402, Sharn Veterinary In, Tampa, FL 33618) was available, and was used intermittently at the discretion of the anesthesiologist, but the values were not consistently recorded. Each anesthetist was instructed to notify the anesthesiologist if the Sp0_2_ < 91% or MAP was < 65 mmHg. The initial protocol did not include IV catheters or endotracheal tubes for either dogs or cats, with the plan that if any dog or cat needed ventilatory support, they would have an endotracheal tube placed. However, following the first day, the anesthesiologist changed the protocol so that all dogs would have an IV cephalic catheter aseptically placed to facilitate IV drug administration and an endotracheal tube placed to assist ventilation with an Ambu bag as needed. Ventilation was assisted via Ambu bag if SpO_2_ < 91%. Any patient that was insufficiently anesthetized before or during surgery, was administered alfaxalone (1.0 mg/kg). Cats were administered alfaxalone IM whereas dogs were administered alfaxalone IV. Respiratory rates and SpO_2_ were evaluated in all cats before beginning of surgery to determine if an endotracheal tube was required (if SpO_2_ < 91%). All patients were routinely shaved and aseptically prepared for surgery. All surgeries were performed by a DVM. Up to 4 animals were sterilized simultaneously, on separate tables at least 2.5 m apart. Each surgeon determined if the patient was pregnant, and that data recorded. Any patient that did not have spontaneous movement following extubation was administered atipamezole. Any dog or cat showing evidence of dysphoria at recovery was administered 0.01 mg/kg acepromazine IM or IV, at the discretion of the anesthesiologist. All patients were observed until fully recovered (sternal recumbency with head up). Time from injection of anesthetic to start of surgery, duration of surgery, total patient time (from injection of anesthetic drugs to full recovery), and recovery rectal temperature were recorded for each patient.

## Data Availability

The datasets used and/or analyzed during the current study are available from the corresponding author on reasonable request.

## Abbreviations

CF: Female cat
CM: Male cat
DF: Female dog
DM: Male dog
SQ: Subcutaneous
OVH: Ovariohysterectomy
IM: Intramuscular
IV: Intravenous
HR: Heart rate
RR: Respiratory rate
SpO_2_%: Saturation of hemoglobin by oxygen
PK/PD: Pharmacokinetics/ pharmacodynamics
DVM: Doctor of Veterinary Medicine
CNS: Central nervous system
